# Unaltered sequence of dental, skeletal, and sexual maturity in domestic dogs compared to the wolf

**DOI:** 10.1186/s40851-016-0055-2

**Published:** 2016-08-22

**Authors:** Madeleine Geiger, Karine Gendron, Florian Willmitzer, Marcelo R. Sánchez-Villagra

**Affiliations:** 1 Paläontologisches Institut und Museum, Universität Zürich, Zürich, Switzerland; 2 Departement für klinische Veterinärmedizin, Vetsuisse-Fakultät, Universität Bern, Bern, Switzerland; 3 Departement für Kleintiere, Bildgebende Diagnostik, Vetsuisse-Fakultät, Universität Zürich, Zürich, Switzerland; 4 Paläontologisches Institut und Museum, Universität Zürich, Zürich, Switzerland

**Keywords:** Tooth, Eruption, Growth plate, Epiphyseal fusion, Maturity

## Abstract

**Background:**

It has been hypothesised that domestication altered the sequence of dental, skeletal, and sexual maturity of dogs when compared to their wolf ancestor. To test this we investigated a comprehensive sample of domestic dogs.

**Methods:**

We documented the timing of completed eruption of permanent dentition into occlusion (dental maturity) and the timing of growth plate closure at the proximal humerus (skeletal maturity) in ontogenetic series of wolves and 15 domestic dog breeds. Data for 137 domestic dog and 64 wolf individuals were collected based on radiographs and examination of macerated bones.

**Results:**

Our analyses show that domestic dogs exhibit a similar sequence of dental and skeletal maturity as the ancestral wolf. Although the absolute change of the age at attainment of sexual maturity is great in domestic dogs as compared to the wolf, the sequence of dental, skeletal, and sexual maturity is not altered as extensively, contradicting one previous hypothesis. Moreover, our data suggest that the chondrodystrophic dachshund attains skeletal maturity earlier than the non-chondrodystrophic breeds examined here.

**Conclusions:**

Domestic dogs are more wolf-like in terms of the sequence of dental, skeletal, and sexual maturation than previously hypothesised. This implies that the domestication process and/or breed formation did not have a major impact on this sequence, although the absolute values of life history variables do have a greater range of variation than in the wild wolf.

**Electronic supplementary material:**

The online version of this article (doi:10.1186/s40851-016-0055-2) contains supplementary material, which is available to authorized users.

## Background

Wolves living as commensals of humans at the beginning of the domestication process experienced changes in environmental conditions [1–3]. These changes may have included unpredictability of food supplies and decreasing interspecific competition, leading to accelerated maturation and increased fecundity [1–3]. Early domestic dogs are therefore hypothesised to have been smaller and to have attained sexual maturity earlier than wolves [3]. It has also been proposed that, as a consequence, the sequence of somatic and sexual maturity was shifted in domestic dogs compared to wolves [4]. Specifically, in wolves dental maturity (completed emergence of the permanent dentition into occlusion) is followed by skeletal maturity (closure of the growth plates in long bones), which is then followed by sexual maturity [4]. In contrast, in domestic dogs it has been proposed that sexual maturity is attained earlier than skeletal and dental maturity, but the sequence of the latter two to one another is not shifted otherwise [4]. The early shift of sexual maturity with respect to dental and skeletal maturity in domestic dogs would also be expected, because uncertain nutritional supply does not affect the skeleton and teeth as greatly as it does sexual maturity [5]. The sequence of dental, skeletal, and sexual maturity in wolves, and changes of this sequence in domestic dogs, have to our knowledge not yet been investigated and reported in any study. Our study therefore aimed at testing whether this sequence is altered in domestic dogs compared to wolves, using a comprehensive dataset.

## Methods

Our sample population included 137 domestic dogs (15 breeds) and 64 wolves; for the determination of the timing of dental maturity, dry skulls of 81 domestic dogs (11 breeds, Table 1) and 42 wolves were used; for the determination of the timing of skeletal maturity, radiographs of 56 domestic dogs (6 breeds, Table 1) and dry bones of 22 wolves were used. Dog breeds were chosen to represent a wide range of body size and proportions (Table 1).

**Table 1:** Shoulder height, body proportions, and number of investigated specimens per domestic dog breed

Dental maturity			Skeletal maturity			
Breed	Shoulder height (cm)	*n*	Breed	Shoulder height (cm)	Body proportions	*n*
Bernese mountain dog	58 – 70	16	Beagle	33 – 41	NCD	6
Boxer	53 – 63	5	Bernese mountain dog	58 – 70	NCD	28
Fox terrier	33 – 41	4	Chihuahua	15 – 23	NCD	5
French bulldog	30	8	Dachshund	20 – 27	CD	7
German shepherd	55 – 65	10	English bulldog	31 – 40	NCD	7
Golden retriever	51 – 61	6	Standard poodle	45 – 60	NCD	3
Great Dane	71 – 86	9				
Leonberger	65 – 80	5				
Newfoundland	63 – 74	8				
St. Bernard	65 – 90	6				
Standard poodle	45 – 60	4				

*CD* chondrodystrophic, *NCD* non-chondrodystrophic, *n* number

Only specimens with a reported individual age of less than 14 months were used since domestic dogs and wolves are generally fully grown towards the end of their first year [6–14]. In the case of the domestic dogs and captive wolves, the age data were sourced from databases provided by the corresponding pet owners and zoological gardens. Individual ages of the wild wolves were approximated using information provided by cementum annuli counts and known breeding seasons and gestation times; estimates of the age of all wolves (in years) are based on cementum annuli counts [15] conducted by the Naturhistoriska Riksmuseet, Stockholm, where the wild wolves used in the present study are housed. We used these age estimates in association with an approximated date of birth: wolves in Finland, at about the same latitude as the middle part of Sweden (about 60°N), where most Swedish wolves used in this study come from, were found to have breeding season in March [16, 17]. Adding a gestation time of 63 days, which is the average in wolves [17–23], the birth of the puppies falls within May. According to the birth records of 10 litters of captive wolves in Berne, Switzerland (47°N), the mean birth date is the 8 May. Because the breeding season, and thus the date of whelping, gets later with increasing latitude [16, 17], the average birth date of the Swedish wolves is probably slightly later than that observed in Swiss wolves. Thus, for the sake of simplicity, we suggest the 15 May as the average birth date of the Swedish wolves. The age of the wolves from Naturhistoriska Riksmuseet was thus estimated as the cementum annuli count plus the difference between the 15 May and their date of death. This approach implies an error of plus/minus about a half a month for calculations of completed permanent dentition into occlusion as well as of closure of the proximal humeral growth plate. Individual ages in all specimens in this study were usually assessed in days and subsequently converted to months. Specimens exhibiting observable pathologies or specimens for which pathology affecting limb bones or the skull was reported in collection or patient databases were not included. Only specimens with a complete set of teeth were included, i.e., specimens with missing or reduced teeth were not considered.

Dry bones and skulls from the following institutions were examined to categorise status of dental eruption in domestic dogs and the wolf as well as of growth plate closure in the wolf: Naturhistorisches Museum Bern (NMBE); Naturhistoriska Riksmuseet, Stockholm (NRM); Zoologisches Museum der Universität Zürich (ZMUZH). Radiographs from the Vetsuisse-Fakultät Bern (VSB, Departement für klinische Veterinärmedizin) were examined to assess the investigation of growth plate closure in domestic dogs. Radiographs had been taken with a computed radiography system (CR, Fuijifilm, Dielsdorf, Switzerland) in the course of medical diagnostic processes in accordance to standard hospital procedures, in standard positions [24]. Our investigations using these radiographs were thus retrospective.

**Fig. 1:**
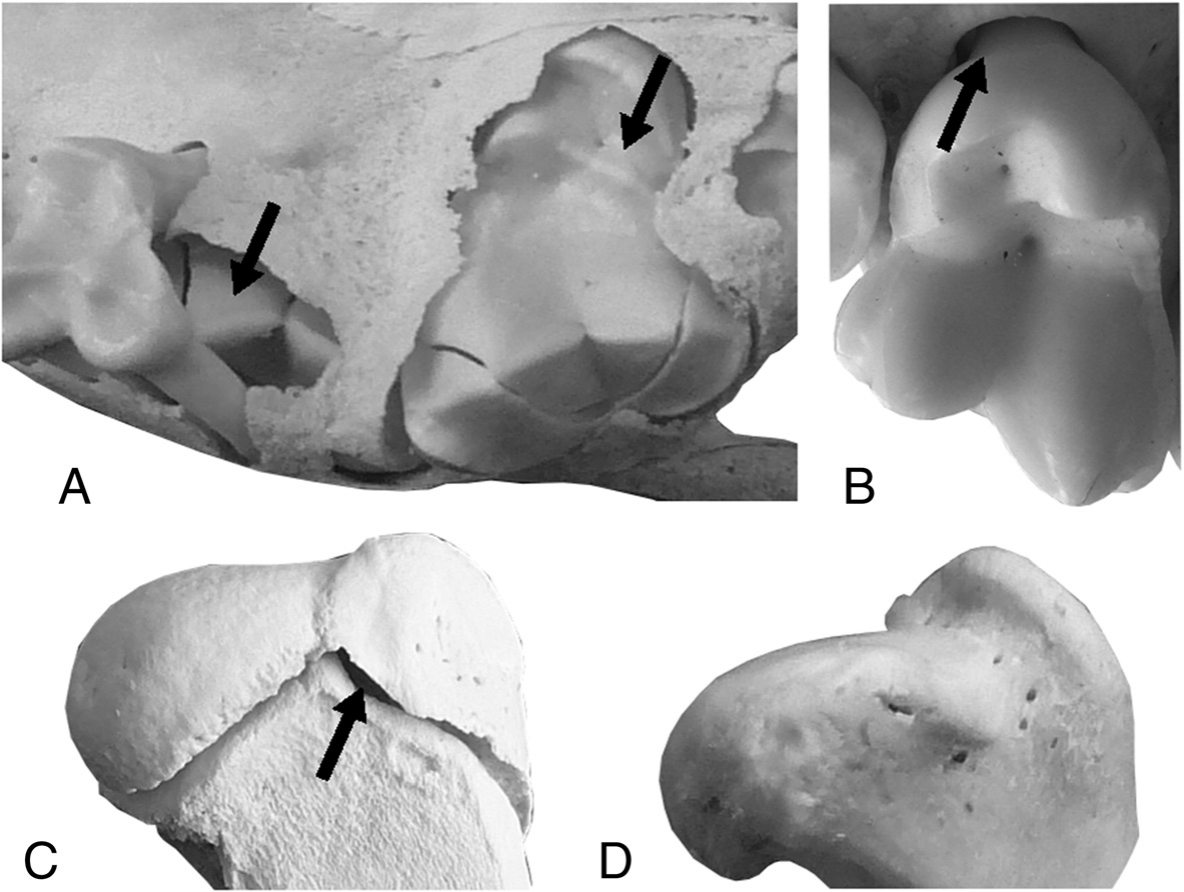
Tooth eruption (**a, b**) and growth plate closure (**c, d**) stages. **a** Stage 1 (left arrow), unerupted, the tooth is not visible above the alveolus; stage 2 (right arrow), erupting, the tooth crown is visible above the alveolus, but not yet in occlusion. **b** Stage 3, erupted, the tooth is in the occlusal plane and the enamel-cementum junction (arrow) is visible above the alveolus. **c** Stage 0, open growth plate (arrow). **d** Stage 1, closed, outer surface is at least in part obliterated by bone

**Fig. 2:**
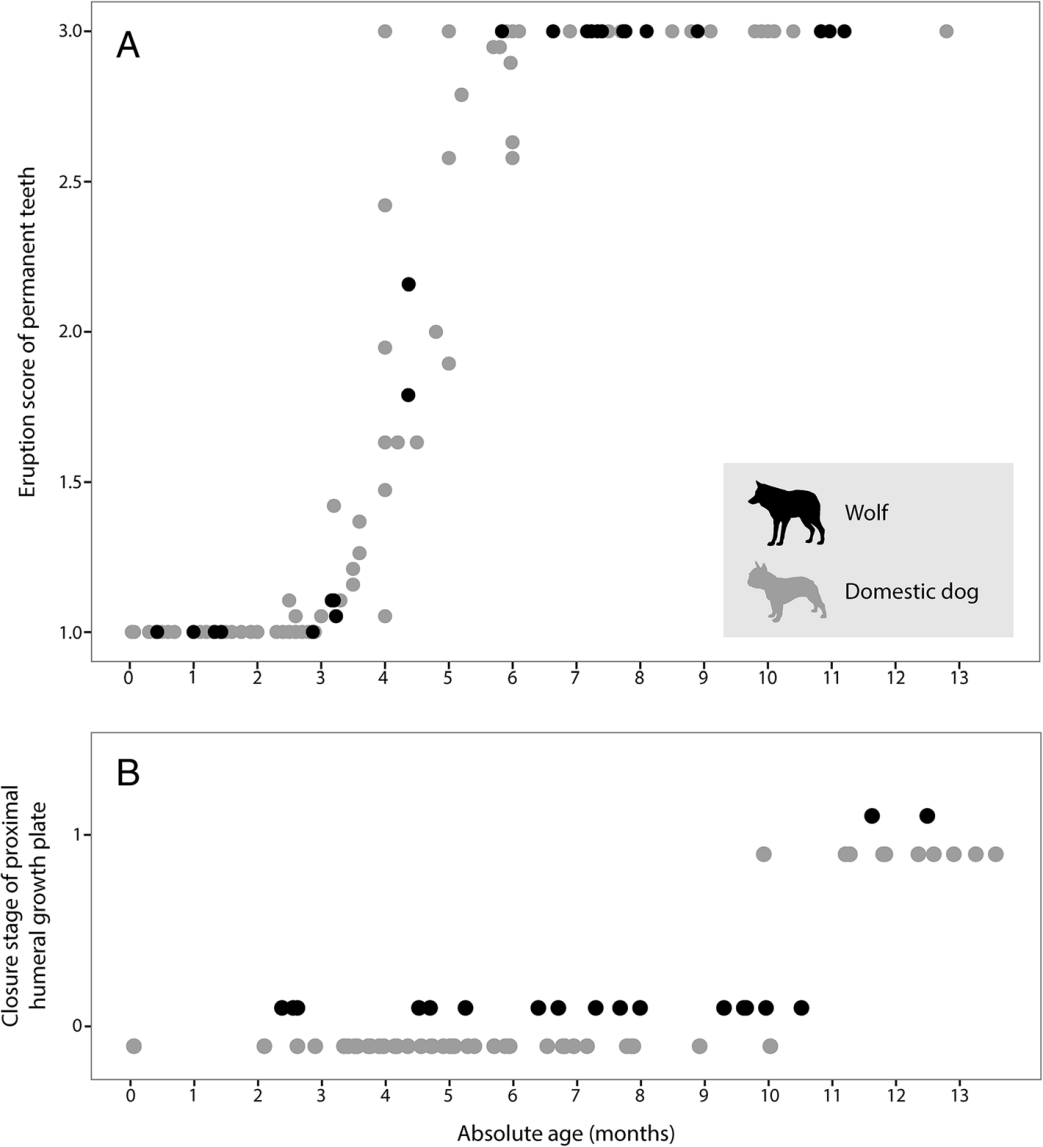
Stages of tooth eruption (**a**) and proximal humeral growth plate closure (**b**) in the investigated wolves and domestic dogs of different absolute ages. The data suggest that dental maturity (**a**) is attained at 4 – 6 months in wolves and domestic dogs and skeletal maturity (**b**) is attained at 10 – 12 months in wolves and 10 – 11 months in domestic dogs (only non-chondrodystrophic breeds). There are no extensive differences between dogs and wolves. Eruption scores (**a**) were calculated as the sum of the eruption stages of all teeth (1, 2 or 3; Fig. 1) divided by the total number of examined teeth. The resulting eruption score thus lays between 1 (all permanent teeth are in stage 1) and 3 (dental maturity attained: all permanent teeth fully erupted in stage 3). Proximal humeral growth plates were scored as either open (stage 0) or closed (stage 1, Fig. 1). Raw data are provided in Additional file 1

The eruption stage of each tooth in the maxilla and the mandible was coded as described by Tappen et al. [25]: 1) unerupted, the tooth is not visible above the alveolus (Fig. 1a); 2) erupting, the tooth crown is visible above the alveolus, but not yet in occlusion (Fig. 1a); 3) erupted, the tooth is in the occlusal plane and the enamel-cementum junction is visible above the alveolus (Fig. 1b). Dental maturity was defined to be attained in a specimen if all incisors, premolars, and molars were fully erupted into the occlusal plane (stage 3 in all teeth). Additionally, the relative amount of eruption (eruption score) was calculated for every specimen: the sum of closure stages (1, 2 or 3) in all teeth was divided by the total number of teeth in the upper and the lower jaw (here 19). The resulting eruption score for every specimen thus lies between 1 (all permanent teeth are in stage 1) and 3 (dental maturity attained: all permanent teeth are in stage 3).

Closure of the proximal humeral growth plate was used as an indicator of skeletal maturity being the last postcranial plate to fuse in the comprehensive examination of the postcranium in wolves (Additional file 1). Each growth plate was scored as either open (stage 0, Fig. 1c) or closed (stage 1, Fig. 1d). Growth plates were considered closed in dry bones if the outer surface of the plate was at least in part obliterated [26]. On radiographs growth plates were considered closed if the radiolucent (black) line between epiphysis and metaphysis was no longer visible in at least one portion of the growth plate [27, 28]. Age at attainment of female sexual maturity was derived from the literature [29–33]. Only females were considered due to the better availability of data on sexual maturity. Details on the specimens and discussion of methods are given in Additional file 1 and Additional file 2.

The here used ontogenetic series served to deduce age ranges at attainment of dental and skeletal maturity in domestic dogs and wolves [34, 35]. The estimated age ranges at (1) completed emergence of dentition into occlusion, (2) closure of the proximal humeral growth plate, and (3) attainment of sexual maturity were compared to one another in the domestic dogs and the wolf, respectively.

There has been disagreement as to whether there are breed specific differences of the age at attainment of dental, skeletal, and sexual maturity within domestic dogs (for details see Additional file 2). Specifically, it has been argued that dental maturity is correlated with breed [36] and body size [37–43]; it has been hypothesised variously that skeletal maturity is correlated with breed [44, 45] nutritional condition [37, 46], sex [29, 44, 47–51], age at time of neutering of puppies [28], and relative limb proportions [27, 44, 52, 53], i.e., chondrodystrophy (disturbed cartilage and endochondral bone growth, resulting in disproportionately short, thickened, and curved long bones [54–56]); sexual maturity has been argued to be correlated with body size [29, 57–61], breed, and housing conditions [57, 58]. Our sampling included domestic dogs of a great range of body sizes (Table 1). Further, both sexes were included and neutered specimens were not considered. Moreover, our domestic dog sample contained only specimens from a middle European origin and we thus considered the living environment rather homogenous. Therefore, many of the variables that might affect the age at attainment of dental and skeletal maturity are unlikely to have biased our estimates. Nevertheless, the influence of two variables on the estimates has been tested here. First, it has been tested whether chondrodystrophy is correlated with the age at attainment of skeletal maturity. Our sample contained one chondrodystrophic breed, the dachshund [62, 63] (Table 1). We examined whether the attainment of skeletal maturity in this chondrodystrophic breed is different from non-chondrodystrophic breeds. Second, we tested whether differences in body size are correlated with the age at attainment of sexual maturity. The here used records of the mean age at attainment of sexual maturity and adult female body weight in 43 domestic dogs breeds ranging from around 3 kg (Affenpinscher) to around 68 kg (St. Bernard) were obtained from Johnston et al. [29]. To determine if there is a correlation between body size and age at attainment of sexual maturity, a non-parametric Spearman’s correlation was calculated using R version 2.15.1 and RStudio version 0.98.501. Since body size was found to be not correlated with the estimate of attainment of sexual maturity (see Results), no corrections of our data was conducted in this regard.

## Results

Our data suggest that in wolves, dental maturity is attained at 4–6 months (Fig. 2a, Additional file 1) and skeletal maturity at 10–12 months (Fig. 2b, Additional file 1). On the basis of examinations of hormone levels, follicle development, and condition of uteri, it has been stated that reproduction in wolves usually does not commence before the 22 month of postnatal life [31, 32]. Reproductive activity of wolves in the first year of life has, however, been reported, but seems to occur only occasionally [14, 30, 33]. An extremely late onset of reproduction (five or six years) is also known to occur [14, 64].

We found evidence that in domestic dogs, dental maturity is attained at 4–6 months (Fig. 2a, Additional file 1) and skeletal maturity at 10–11 months (only if considering non-chondrodystrophic breeds, see below) (Fig. 2b, Additional file 1). The age at attainment of sexual maturity ranges from around seven months (border collie, Cavalier King Charles spaniel) to 21 months (Italian greyhound), with a mean of about 12.5 months across all reported breeds [29].

There was a non-significant correlation between mean adult female body mass and age at attainment of sexual maturity (r_S_ = 0.064, *P* = 0.682). We found evidence that the attainment of skeletal maturity in the chondrodystrophic dachshund occurs at 8–9 months (Additional file 1), an age at which none of the here investigated non-chondrodystrophic domestic dogs exhibited a closed proximal humeral growth plate and thus skeletal maturity (Additional file 1). The dataset supporting this article has been uploaded as part of the supplementary information (Additional file 1).

## Discussion

Estimated age at attainment of dental maturity in domestic dogs and wolves as determined in this study (4–6 months) is in agreement with previous reports of values ranging from 6 to 7 months in the German Shepherd [39], 5.4–8.4 months in the bull terrier [43], and 6–7 months in wolves [14]. Slightly lower estimates in this study compared to the literature are probably the consequence of the exclusion of the canine teeth (Additional file 2). Our estimates of age at attainment of skeletal maturity in domestic dogs (10–11 months) and the wolf (10–12 months) are in agreement with published ranges of 8.5 to 13.75 months in the domestic dog [27, 44, 45, 47, 48, 50, 51, 65–67] and 10.8 to 12.3 months in the wolf [50], respectively. Our results show that there is no shift of the sequence of attainment of skeletal maturity in respect to dental maturity in domestic dogs compared to wolves. This finding substantiates previous hypotheses [50, 68]. We would like to point out here that the general applicability of our results is restricted by the sample, which is limited due to several factors: first, due to a lack of associated skulls and postcranial skeletons, the age estimates for dental and skeletal maturity are based on different specimens and joining of breeds (see also Additional file 2); second, the different extant ‘subspecies’ of the wolf were not considered for this study (Additional file 1) since it is literally impossible to sample the original population(s) of wolves which was (were) domesticated [69]. Third, our methodology does not allow for continuous data that can be easily statistically tested (see below).

It has been discussed if the age at attainment of skeletal maturity is associated with chondrodystrophy, a congenital disturbance which affects mainly the cartilage and has a negative impact on the growth of endochondral bone, and thus also long bones of limbs [27, 44, 52, 53]. Our result supports the view that the age at attainment of skeletal maturity in the chondrodystrophic dachshund is attained earlier (8–9 months) than in non-chondrodystrophic breeds. However, the early attainment of skeletal maturity in the chondrodystrophic breed does not alter the sequence of dental and skeletal maturity in this breed. Further, there is evidence for a relationship between epiphyseal growth plate closure and the attainment of sexual maturity. It has been found that oestrogen treatment accelerates senescence of growth plates in ovariectomised rabbit tibiae, leading to early growth plate closure relative to untreated ovariectomised specimens [70]. Effects of oestrogen on growth plate physiology have also been found in other species (for a review see [71]). Similarly, as described above, it has been shown that gonadectomy, and thus potentially lower levels of oestrogens, delays growth plate closure in domestic dogs [28]. As oestrogen is crucial for the development of sexual organs, the attainment of skeletal and sexual maturity may be interrelated by means of this hormone.

It has been hypothesised that in wild canids, dental maturity and skeletal maturity were attained before sexual maturity, with dental maturity preceding skeletal maturity (dental – skeletal – sexual) [4]. In domestic dogs, this sequence was hypothesised to have reversed to sexual – dental – skeletal due to changed environmental conditions during domestication [4]. Our findings support this hypothesis for the wolf: dental maturity is attained at 4–6 months, followed by skeletal maturity at 10–12 months, and finally sexual maturity at about 22 months on average. However, it is important to note that in some wolves, skeletal and sexual maturity might overlap. This overlap could for example occur in wolves that attain sexual maturity with nine or ten months (see below) [14, 30, 33]. Domestic dogs have been found to attain dental and skeletal maturity at a similar age as wolves, but the age at attainment of sexual maturity is generally lower than in wolves (7–21 months). It has therefore to be expected that the sequence in domestic dogs with comparably late attainment of sexual maturity is similar as in wolves (dental – skeletal – sexual). In other breeds with an average or low age at attainment of sexual maturity, on the other hand, there might be a heterochronic shift of sexual maturity in respect of skeletal maturity (dental – sexual – skeletal), or sexual and skeletal maturity occur simultaneously (dental – skeletal/sexual). According to our estimates it is, however, unlikely that sexual maturity would ever be attained before the attainment of dental maturity in domestic dogs.

Reasons for the relatively early attainment of sexual maturity in domestic dogs compared to wolves may lie in the social system of these two forms. Due to suppression of sexual maturity in subordinate pack members through dominance of the alpha-female, young wolves breed mostly only after they disperse from their natal pack, at 11–24 months of age or later [72]. In domestic dogs there is no such reproduction control, and sexual maturity is attained within the first year or up to 1.5 years [73]. The early onset of reproduction in domestic dogs might thus be the result of the absence of a complex social system, such as that found in wolves, and a loss of susceptibility to social suppression [30, 74, 75]. This latter element has been speculated to be based on the close proximity of domestic dogs to humans, with subsequent ample opportunities to scavenge, which has made the dependence of domestic dogs on one another, and thus monogamy and true pack behaviour, unnecessary [74].

## Conclusions

While we detected no change in the age at attainment of dental and skeletal maturity in domestic dogs as compared to wolves, the change in the absolute age at the attainment of sexual maturity is extensive. Although the relatively early attainment of sexual maturity in domestic dogs compared to wolves signifies a change in the hypothesised direction, it does not result in a sequence change of dental, skeletal, and sexual maturity as extensive as previously hypothesised, with sexual maturity preceding dental and skeletal maturity in domestic dogs [4]. Preservation of life history variables within species and clades has been reported [76] and our study substantiates this finding.

With the sample at hand it is not possible to corroborate that the observed pattern has been established during the early domestication process or is the result of the more recent establishment of modern breeds. Future research on skeletal and dental maturity in dogs during the early domestication process will require ontogenetic series of fully articulated prehistoric specimens. Alternatively, pariah dogs could serve as an approximation for domesticated dogs during the early domestication process. Pariah, or stray, dogs, are ownerless dogs that depend indirectly on humans for food and shelter [74]. As such, they are human commensals, i.e., they take advantage of elements of the human niche, and therefore mirror the commensal relationship that has been suggested for humans and wolves/domestic dogs during the early domestication process [77, 78]. To set the research on the attainment of somatic and sexual maturity in a much wider context, more domesticated species should be considered. For example, the examination of penned pigs versus free ranging pigs versus wild boar would be worthwhile (as an example see Evin et al. [79]). Especially useful would be the investigation of species that have been domesticated relatively recently and were subject to a human directed, immediate, and rapid form of domestication, using established knowledge about previous domestication processes (‘directed’ pathway) [78]. In such species, e.g. golden hamster (*Mesocricetus auratus*), the ancestral wild populations are likely to be better known and extant, unlike those for some ‘ancient’ domesticates, such as dogs. Moreover, the examination of domesticates of various body sizes would make possible the investigation of the order of dental, skeletal, and sexual maturity in relation to other life history traits, for example longevity, a kind of study also applicable to island forms and their known similar pattern of evolution to domesticated forms [80]. It has been suggested that species with a relatively short lifespan, e.g., small rodents, attain sexual maturity only after dental and skeletal maturity (dental – skeletal – sexual), whereas sexual maturity is attained earlier than dental and skeletal maturity (sexual – dental – skeletal) in mammals with a relatively long lifespan, e.g. great apes [4, 81]. Although this pattern was suggested to be only valid for wild species, and not for domestic forms, these relationships require more empirical tests. Finally, studies on the investigation of somatic versus sexual maturity would benefit from an improved methodology. This would include a longitudinal growth series of domestic dogs and wolves, for which CT-scans of the same individuals would be produced throughout their development from birth to adulthood, maybe also under different environmental conditions (e.g., food supply, nutritional content of food). These CT-cans could be used to measure percentages of growth plate fusion and tooth eruption and therefore provide, together with measures of total bone length and total tooth size, better continuous data to be tested statistically. Such an extensive study was beyond the possibility of the project presented here and may not yield different results.

## Additional files

Additional file 1: Tooth eruption and growth plate closure stages in the wolves and domestic dogs examined in this study. (XLSX 34 kb) Additional file 2: Details and discussion on data acquisition, and variation of dental, skeletal, and sexual maturity within domestic dogs. (DOCX 20 kb)

## Abbreviations

CD: Chondrodystrophic
NCD: Non-chondrodystrophic
NMBE: Naturhistorisches Museum Bern, Switzerland
NRM: Naturhistoriska Riksmuseet, Stockholm, Sweden
VSB: Vetsuisse-Fakultät Bern (Departement für klinische Veterinärmedizin), Switzerland
ZMUZG: Zoologisches Museum der Universität Zürich, Switzerland
