# Supplementary material for: Unaltered sequence of dental, skeletal, and sexual maturity in domestic dogs compared to the wolf
Source: Zoological Lett. 2016 Aug 22;2(1):16. doi: 10.1186/s40851-016-0055-2 (PMC4994403; doi:10.1186/s40851-016-0055-2)
Supplement: Additional file 2: — Details and discussion on data acquisition, and variation of dental, skeletal, and sexual maturity within domestic dogs. (DOCX 20 kb) [file 40851_2016_55_MOESM2_ESM.docx]

**Supplementary Information**

**Details and discussion of data acquisition**

***Dental maturity***

The time that elapses among the coded stages of eruption is variable. This is apparent on the wolf example. While stage 1 lasts about 3 to 4 months, stage 2 lasts about 1 month (**Additional file 1**). The canine teeth were not considered because it is difficult to determine completed eruption in many domestic dog specimens because the alveolar bone around the canine teeth is prominent and obscures the enamel-cementum junction.

***Skeletal maturity***

There are two limitations concerning the study of growth plate closure as exerted here. First, we compare age estimates at the attainment of skeletal maturity using dry bones (wolf) and radiographs (domestic dogs). In their study about tooth eruption and growth plate fusion in domestic pigs and wild boar, Bull and Payne [1] mentioned that it is not clear whether data about growth plate closure obtained through observing dry bones and radiographs are comparable. On the other hand, Harris [2] reported that growth plate closure in red foxes recorded by using radiographs and by examining dry bones yielded the same results.

The main problem in comparing data from dry bones and radiographs is the pattern of growth plate closure: the ossification starts in the central parts of the growth plate and subsequently extends to the outer surface [3]. If the central part of the growth plate starts to ossify, these parts of the growth plate appear as radiopaque areas in a radiograph. Such a growth plate would be considered as closed if the definition of growth plate closure used in this study is applied. We chose this definition because the bone does not grow any longer in length as soon as parts of the growth plate are ossified [4]. The definition of growth plate closure in dry bones used in this study is based on the obliteration of the outer surface of the bone. We chose this definition because in dry bones beginning closure in the centre cannot be determined. Very often it cannot be distinguished if an epiphysis or an apophysis is just glued to the metaphysis or actually already slightly ossified. Therefore, the age at closure of the growth plates using dry bones might be overestimated compared to estimates using radiographs. The second limitation concerning the study of growth plate closure as exerted here applies to the radiographs used for this study. Since most radiographs were taken during standard diagnostic procedures, they often display left lateral views of the thoracic region. Due to the thoracic focus of these images, the humerus is on the outer rim and additionally superimposed with the contralateral humerus and possible other items, such as endotracheal tubes. In cases where the stage of closure was not determinable without doubt, the radiograph in question was therefore double checked and re-evaluated by more than one of the authors. Additionally, there is one shortcoming of radiographs, which is that variability in energy and exposure time can result in structures being either undetected or overexposed and thus not visible on the radiograph.

## Variation of dental, skeletal, and sexual maturity within domestic dogs

Within domestic dogs it has been disagreement as to whether there are breed specific differences of the age attainment of dental, skeletal, and sexual maturity. Many authors have reported breed specific differences of the timing of tooth eruption: Scott and Fuller [5] reported delayed completed eruption of the upper deciduous canine teeth in the fox terrier compared to basenji, beagle, cocker Spaniel, and Shetland sheepdog. Huidekoper [6] noted that the permanent teeth erupt several months earlier in large breeds compared to smaller terriers. Medium sized breeds (e.g., setters) are in an intermediate position. Similar statements were made by other authors [7-10]. However, none of these authors presented data to support these statements. Evans [11] went even further, stating that the life span of a breed correlates with the timing of tooth eruption: short lived, large breeds’ teeth erupt earlier than long lived, small breeds’ teeth. Similar statements have also been provided by several authors as described in Arnall [12]. Any influence of sex on the timing of tooth eruption has not yet been reported.

The sequence of growth plate closure has been reported to be constant among domestic dog breeds [13], but whether the absolute timing of closure of the growth plates is different among domestic dog breeds has been a matter of debate. Sumner-Smith [14]found that there are no differences among poodle, greyhound, and German shepherd, whereas Hare [13,15] reported differences of the timing of growth plate closure among breeds. Some degree of variation has been reported among specimens of the same breed [13,16-18] and even among litter mates in the red fox [2], the wolf [19], and the domestic dog [13,14]. Age at growth plate closure is further expected to depended on nutritional conditions [20] and age at time of neutering of puppies [21]. The influence of sex on growth plate closure was reported to be not significant [13,14,21-24], whereas only one study argued that there is some variation due to sex [4]. It has further been discussed whether the age at attainment of skeletal maturity is associated with chondrodystrophy [13,25-27]. Chondrodystrophy is a congenital disturbance which affects mainly the cartilage and has a negative impact on the growth of endochondral bone, resulting in disproportionately short, thickened, and curved long bones [16,28,29].

Among breeds of domestic dogs, females of small breeds are supposed to attain sexual maturity earlier than females of larger breeds [30-35], although this correlation has also been debated [34]. The age at sexual maturity may also be affected by line and breed dependent genetic factors, cross-breeding, diet, and housing conditions [30,31].

**Supplementary references**

1. Bull G, Payne S (1982) Tooth eruption and epiphysial fusion in pigs and wild boar. In: Wislon B, Grigson C, Payne S, editors. Aging and sexing animal bones from archaeological sites. Oxford British Archaeological Reports. pp. 55-71.

2. Harris S (1978) Age determination in the red fox (Vulpes vulpes) - an evaluation of technique efficiency as applied to a sample of suburban foxes. J Zool 184: 91-117.

3. Todd TW (1930) The anatomical features of epiphysial union. Child Dev 1: 186-194.

4. Smith RN, Allcock J (1960) Epiphysial fusion in the greyhound. Vet Rec 72: 75-79.

5. Scott JP, Fuller JL (1965) Genetics and the social behavior of the dog. Chicago: University of Chicago Press.

6. Huidekoper RS (1891) Age of the domestic animals: being a complete treatise on the dentition of the horse, ox, sheep, hog, and dog, and on the various other means of determining the age of these animals. Philadelphia, London: F. A. Davis.

7. 18. Bell AF (1965) Dental disease in the dog. J Small Anim Pract 6: 421-428.

8. Habermehl K-H (1975) Die Altersbestimmung bei Haus- und Labortieren. Berlin: Parey.

9. Liautard A (1885) How to tell the age of domestic animals. New York: William R. Jenkins, Veterinary Publisher and Bookseller.

10. Wehrend A (2013) Neonatologie beim Hund – Von der Geburt bis zum Absetzen. Hannover: Schlütersche Verlagsgesellschaft.

11. Evans HE (1993) Miller's Anatomy of the Dog. Philadelphia: Saunders.

12. Arnall L (1960) Some Aspects of Dental Development in the Dog—II. Eruption and Extrusion. J Small Anim Pract 1: 259-267.

13. Hare WC (1960) The age at which epiphyseal union takes place in the limb bones of the dog. Wiener Tierärztliche Monatsschrift, Festschrift Schreiber: 224-243.

14. Sumner-Smith G (1966) Observations on epiphyseal fusion of the canine appendicular skeleton. J Small Anim Pract 7: 303-311.

15. Hare WCD (1959) Radiographic anatomy of the canine pectoral limb. II. Developing limb. J Am Vet Med Assoc 135: 305-310.

16. Kealy JK, McAllister H, Graham JP (2011) Diagnostic radiology and ultrasonography of the dog and cat. St. Louis: Saunders.

17. Weise G (1966) Über das Wachstum verschiedener Haushunderassen. Z Säugetierkd 31: 257-282.

18. Chapman WL (1965) Appearance of ossification centers and epiphysial closures as determined by radiographic techniques. J Am Vet Med Assoc 147: 138-141.

19. Van Ballenberghe V, Mech LD (1975) Weights, growth, and survival of timber wolf pups in Minnesota. J Mammal 56: 44-63.

20. Wayne HR, Shirer JF (1965) Normal and abnormal growth of the distal foreleg in large and giant dogs. Vet Radiol Ultrasound 6: 50-64.

21. Salmeri KR, Bloomberg MS, Scruggs SL, Shille V (1991) Gonadectomy in immature dogs: effects on skeletal, physical, and behavioral development. J Am Vet Med Assoc 198: 1193-1203.

22. Schroeder M (1978) Beitrag zur Entwicklung des Skelettes der Vordergliedmasse beim Deutschen Schäferhund [Dissertation]. München: Ludwig-Maximilians-Universität München.

23. Widmer W (1978) Beitrag zur Entwicklung des Skeletts der Hintergliedmasse beim Deutschen Schäferhund [Diss Tiermed Mèunchen, 1978]. München: Ludwig-Maximilians-Universität München.

24. Wiechering G (1981) Untersuchungen über den Epiphysenfugenschluss bei Wölfen und Haushunden. Schriften aus der Archäologisch-Zoologischen Arbeitsgruppe Schleswig-Kiel 5: 1-68.

25. Hitz D (1973) Untersuchungen über Skelett- und Gelenkveränderungen beim Basset-Hound [Dissertation]. Zürich: Universität Zürich.

26. Hanlon GF (1962) Normal and abnormal bone growth in the dog. Vet Radiol Ultrasound 3: 13-15.

27. Owens JM, Biery DN, Hartung K (1989) Röntgenbildinterpretation für den Kleintierpraktiker. Stuttgart: Enke.

28. Jezyk PF (1985) Constitutional disorders of the skeleton in dogs and cats. In: Newton CD, Nunamaker DM, editors. Textbook of Small Animal Orthopaedics. Ithaca, New York: International Veterinary Information Service.

29. Almlöf J (1961) On achondroplasia in the dog. Zentralblatt für Veterinärmedizin 8: 43-56.

30. Cupps PT (1991) Reproduction in domestic animals. San Diego: Academic Press.

31. England GCW (2013) Dog breeding, whelping and puppy care. West-Sussex: Wiley-Blackwell.

32. England GCW, Heimendahl Av (2010) BSAVA manual of canine and feline reproduction and neonatology. Cheltenham: British Small Animal Veterinary Association.

33. Root Kustritz MV (2010) Clinical canine and feline reproduction – evidence-based answers. Iowa: Wiley-Blackwell.

34. Johnston SD, Kustritz MVR, Olson PS (2001) Canine and feline theriogenology. Philadelphia: Saunders.

35. Darwin C (1868) The variation of animals and plants under domestication. Charleston: BiblioBazaar.
